# Effects of exposure to insecticides on sleep and neurobehavioural functioning in puberty and adolescence: a scoping review

**DOI:** 10.2478/aiht-2025-76-4020

**Published:** 2025-09-30

**Authors:** Patricia Tomac, Adrijana Košćec Bjelajac, Ivana Hromatko, Veda Marija Varnai

**Affiliations:** Institute for Medical Research and Occupational Health, Zagreb, Croatia; University of Zagreb Faculty of Humanities and Social Sciences, Zagreb, Croatia

**Keywords:** adolescents, behaviour, carbamates, cognition, emotions, neonicotinoids, organophosphates, pubertal development, pyrethroids, sleep quality, adolescenti, emocije, karbamati, kognicija, kvaliteta spavanja, neonikotinoidi, organofosfati, piretroidi, ponašanje, pubertalni razvoj

## Abstract

Insecticides are pervasive in modern world, with humans being exposed through multiple pathways including dietary intake, occupational exposure, farming activities, residential proximity to crops, and household use. Most commonly used insecticides are neonicotinoids, pyrethroids, organophosphates, and carbamates. Recent evidence suggests that even low-level exposure to these substances may have adverse effects. Adolescence, characterised by intensive maturation processes, is a period of heightened vulnerability to environmental toxicants which may increase the risk of suboptimal developmental outcomes. This review aimed to synthesise the evidence of association between insecticide exposure in childhood/adolescence and sleep and neurobehavioural functioning in children and adolescents aged 8–20 years. Literature search across Web of Science, PubMed, Scopus, and PsycINFO produced 1,492 unique records, of which 48 studies met the inclusion criteria and underwent full-text analysis. Nine of the analysed studies investigated occupational exposure. Most employed cross-sectional design. Insecticide exposure was most often assessed through biomonitoring, questionnaires or interviews, temporal or spatial proximity to crops, and environmental sampling. Occupational exposure studies were primarily conducted in Africa, whereas nonoccupational studies were mostly based in the Americas, Asia, and Europe. Cognitive functioning was the most evaluated aspect of neurobehavioural functioning, while sleep was assessed only in one study. Although the findings are heterogeneous, they suggest that both work-related and residential exposures may affect neurobehaviour and sleep in puberty and adolescence. However, further longitudinal research is needed to clarify causation and also incorporate sleep health and pubertal maturation into the design, both as outcomes and mediators of neurobehavioral effects.

## Abstract (hr)

Insekticidi su sveprisutni u suvremenom svijetu, a ljudi su im izloženi na više načina, uključujući unos hranom, profesionalnu izloženost, poljoprivredne aktivnosti, blizinu stambenih objekata usjevima te kućnu uporabu. Najčešće korišteni insekticidi danas su neonikotinoidi, piretroidi, organofosfati i karbamati. Nedavni dokazi upućuju na to da čak i niska izloženost insekticidima može imati štetne učinke. Adolescencija, obilježena intenzivnim procesima sazrijevanja, razdoblje je povećane osjetljivosti na štetne okolišne čimbenike, što može povećati rizik od nepovoljnih razvojnih ishoda. Cilj ovoga preglednog rada bio je objediniti rezultate istraživanja o učincima postnatalne izloženosti insekticidima na spavanje i neurobihevioralno funkcioniranje tijekom puberteta i adolescencije. Pretraga literature provedena je u bazama *Web of Science, PubMed, Scopus* i *PsycINFO*. Uključene su studije procjene izloženosti insekticidima i neurobihevioralnih ishoda u djece i adolescenata u dobi od 8 do 20 godina. Od 1492 jedinstvena članka, njih 48 zadovoljilo je kriterije uključivanja i analizirani su u cijelosti. U devet od uključenih studija ispitivala se profesionalna izloženost insekticidima. U većini studija koristio se presječni dizajn. Izloženost insekticidima najčešće se procjenjivala pomoću biomonitoringa, a druge metode uključivale su upitnike ili intervjue, vremenski odmak ili prostornu udaljenost od mjesta primjene insekticida te uzorke iz okoliša. Studije profesionalne izloženosti poglavito su provedene u Africi, a studije koje nisu vezane za profesionalnu izloženost uglavnom su se provodile u Americi, Aziji i Europi. Kognitivno funkcioniranje bilo je najčešće ispitivani neurobihevioralni ishod, a spavanje se analiziralo samo u jednoj studiji. Iako su nalazi bili heterogeni, oni upućuju na povezanost između izloženosti insekticidima i štetnih neurobihevioralnih ishoda u pubertetu i adolescenciji. Dokazi sugeriraju da i profesionalna i svakodnevna izloženost insekticidima mogu predstavljati rizik za neurobihevioralno funkcioniranje i spavanje adolescenata. Međutim, potrebno je provesti rigoroznija longitudinalna istraživanja kako bi se ispitala uzročnost te u istraživački nacrt uključile varijable spavanja i pubertalnog razvoja kao ishode, ali i kao potencijalne medijatore neurobihevioralnih učinaka.

Increasing food demands that followed a continuous increase in the world population over decades had led to more intensive use of pesticides in agriculture, including insecticides. The latter are biological agents or natural or synthetic chemical substances used to control the spread of pest insects (1). Several classes of insecticides have been discovered and developed over the years. Currently, the most common in the European Union (EU) and worldwide are neonicotinoids, pyrethroids, organophosphates (OPs), and carbamates. Even adhering to good agricultural practices (2) will not ensure complete removal of their residues from various food products, and ingestion of contaminated food has been identified as the main route of non-occupational human exposure. Other exposure pathways are inhalation or dermal absorption. The latter is especially important in occupational settings.

Many rural economies still heavily rely on child labour, and one of “the worst forms of child labour” is working in environments involving exposure to hazardous substances, such as agrochemicals used in pesticide application (3, 4). As of 2024, over 137.5 million children aged 5–17 are engaged in child labour worldwide, with approximately 61 % of these children working in hazardous agricultural settings (4). This burden is the greatest in Africa, where many children work on small farms or perform household chores to support their families in farming or food production, which includes tasks such as pesticide handling and application (4, 5).

An increasing number of epidemiological studies suggest that exposure to insecticides, even at low concentrations, may adversely affect the nervous and reproductive systems in humans (6,7,8,9,10,11,12,13). Although mammalian nervous systems are generally much less susceptible to the toxic effects of contemporary insecticides due to differences between insects and mammals in body size, temperature, metabolism, detoxification capacities, brain receptors, and ion channel kinetics (which underlie the higher selectivity of certain pyrethroids and neonicotinoids) (14,15,16), most insecticides are not highly selective and may be neurotoxic to humans at chronic, relatively low-level exposure (17). Many insecticides have been also suspected to be endocrine disruptors (18).

Children are especially vulnerable to environmental toxins during sensitive periods of development that occur *in utero*, during infancy, and around puberty (17, 19,20,21). At low levels of exposure, some toxic chemicals from our environment would cause little or no adverse effects in adults yet induce subclinical neurodevelopmental disorders in children. This kind of subclinical neurotoxicity is unlikely to be recorded in public health statistics and is therefore sometimes called a “silent pandemic” (22). Over the past decades, exposure to endocrine-disrupting chemicals has been associated with earlier onset of puberty and an accelerated pubertal development (12, 23, 24), which, in turn, have been associated with adverse physical and mental health outcomes later in life (25).

The onset of puberty, comprising the endocrine processes of adrenarche, gonadarche, and the activation of growth axis, marks the transition from childhood to adolescence and leads to reproductive maturity (26, 27). Gonadarche, often referred to as puberty in the strict sense, involves the activation of the hypothalamic-pituitary-gonadal axis and culminates in the ability to reproduce. In girls, this process usually begins about a year earlier than in boys, occurring between the ages 8–14 (27). Hormonal and neurotransmitter changes during this period trigger a second wave of structural and functional reorganisation in various brain systems and circuits (28, 29) accompanied by changes in behaviour, motivation, and cognitive and socioemotional functioning. Biological systems regulating sleep and circadian rhythms also experience marked changes during puberty: homeostatic sleep pressure accumulates more slowly across the day, and the sleep phase of the circadian rhythm shifts towards later hours (30,31,32,33,34). In average, this phase shift continues until the age of 19.5 years in women and 20.9 years in men (35). After that, the shift reverses toward an earlier sleep phase, which could be considered a potential biological marker of the end of adolescence (35).

Due to the intensity of maturational processes, puberty entails heightened vulnerability to environmental toxicants, including neurotoxic and endocrine-disrupting chemicals, which can lead to suboptimal or adverse developmental outcomes. Besides exposure intensity, the timing is critical, as its effects depend on which structures and functions are developing at that time (20).

Over the past years, researchers have accumulated evidence linking prenatal exposure to commonly used pesticides with adverse neurodevelopmental outcomes in children and adolescents. However, evidence regarding the effects of exposure during childhood and adolescence remains limited and inconsistent (36). The aim of this scoping review was to take a look at current evidence from studies investigating the effects of childhood/adolescence exposure to insecticides on sleep and neurobehavioural functioning in puberty and adolescence. Our interest was both methodological and theoretical. First, we were interested in the state of research, particularly how exposure and neurobehavioural outcomes were assessed, which confounding factors were controlled for, and whether the stage of pubertal development was considered in the analyses or not. Second, we were interested in analysing the existing evidence and identifying gaps in knowledge by comparing reported non-occupational and occupational exposure, given their typically distinct exposure pathways, frequencies, intensities, and public health implications. We paid special attention to studies conducted in Europe, considering the existing differences in legislation and insecticide use across continents.

The specific research questions that steered our investigation were:

What is the state of research on neurobehavioural outcomes of childhood/adolescence exposure to insecticides in puberty and adolescence?
  1.1. How were the studies designed, which classes of insecticides they included, and how was exposure established?
  1.2. Which neurobehavioural outcomes were investigated, and how were they assessed?
  1.3. Which confounding factors were controlled for in the analyses, and was pubertal development considered?
What evidence exists on the neurobehavioural effects of childhood/adolescence exposure to insecticides in puberty and adolescence?
  2.1. What effects of exposure to insecticides were observed on sleep, cognition, and emotional and behavioural functioning?
  2.2. What are the gaps in knowledge and inconsistencies on these effects?
What are key differences in the methods and findings between studies investigating occupational and non-occupational insecticide exposure among adolescents?
What is the state of research and evidence from studies conducted in Europe?

## METHODS

### Database search and study selection

**Figure 1:**
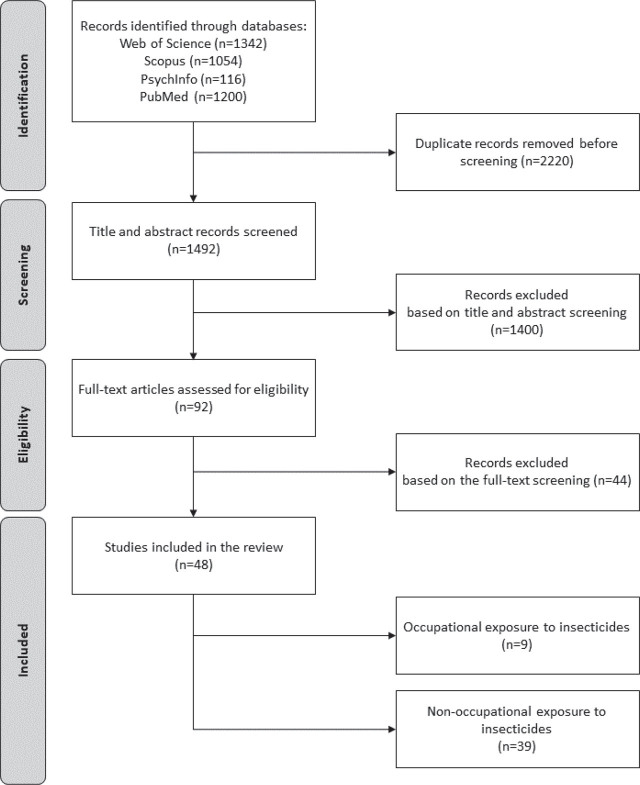
Flow diagram of article search and selection

This scoping review follows the guidelines for Preferred Reporting Items for Systematic Reviews and Meta-Analysis extension for scoping reviews (PRISMA-ScR) (37). Figure 1 details the search and article selection procedures. We applied no date limits, which means that the search included all relevant articles published until 7 January 2025. Literature searches were conducted across Web of Science (WOS; all databases), Scopus, APA PsychInfo, and PubMed. The following search terms were used in the title/abstract fields: (organophosph* OR pyrethroid* OR neonicotinoid* OR carbamate* OR insecticid* OR pesticid*) AND (pubert* OR pubescent OR adolescen* OR teenage* OR “school age” OR child*) AND (sleep* OR circadi* OR chronotype* OR morningness OR eveningness OR “morning type*” OR “evening type*” OR neurobehavi* OR neuropsycholog* OR neurocogniti* OR cogniti* OR “executive function*” OR “executive control” OR inhibit* OR intell* OR memory OR attention OR vigilance OR “reaction time” OR psychomotor OR socioemot* OR emotion* OR “social behavio*” OR “mental health” OR internali* OR externali* OR autism OR “attention deficit” OR hyperactivity OR impulsiv* OR ADHD OR neurodevelop*). Through titles/abstracts in English identified with these key terms, we searched for original research articles and conference papers published in journals and proceedings, focusing on the effects of exposure to insecticides on sleep and neurobehavioural functioning in puberty and adolescence. Articles written in Bosnian, Bulgarian, Croatian, English, French, German, Italian, Russian, Serbian, Serbo-Croatian, and Spanish were considered, as the authors understand these languages sufficiently for full-text analysis.

In total, 1492 unique records were identified across the databases for further screening (Figure 1). Except for publication type and language, no other limitations were applied. To verify the search strategy, two reviewers searched the literature independently. The records retrieved were identical across all databases except Scopus, in which a syntax-based search performed by one reviewer yielded more results than the drop-down menu search performed by the other. For further screening, we, therefore, included articles retrieved through the search method that yielded more results (i.e., syntax-based).

To identify and exclude duplicate records and animal studies and to screen titles and abstracts for eligibility we relied on a web-based, artificial intelligence-powered tool Rayyan (Rayyan Systems, Inc., Cambridge, MA, USA). Studies were included in the review if they met the following criteria: 1) assessment of exposure to organophosphates, pyrethroids, carbamate, and neonicotinoids; 2) evaluation of any aspect of neurobehavioural functioning as an outcome; and 3) outcome assessed at the time when participants were between 8 and 20 years of age, to make sure to capture the entire period of adolescence, beginning with the onset of puberty.

After the exclusion of animal studies (n=166), article screening proceeded in two steps: 1) title and abstract screening, and 2) full-text screening. Initially, the principal author conducted title and abstract screening of all records (n=1492). Subsequently, each of the other three co-authors independently screened a randomly selected subset comprising 33 % of all articles to evaluate accuracy of the title and abstract screening. Inter-rater agreement was not statistically evaluated. All identified conflicts were minor and had been resolved through discussion. Initial screening yielded 92 articles eligible for full-text analysis. Based on the full-text analysis, 44 articles were excluded. We excluded studies that investigated only neurodevelopmental disorders as outcomes (n=26) (e.g., attention deficit disorder, autism spectrum disorder, learning disabilities) because neurobiological disorders typically originate in early childhood, are influenced by complex interaction between genetic and environmental factors, tend to persist throughout life (38, 39), and were therefore not in our scope of interest (short- to medium-term functional neurobehavioural outcomes relevant to puberty and adolescence). We also excluded studies that focused exclusively on prenatal exposure to insecticides (n=13). Additionally, five more studies were excluded due to a wrong aim (n=3) or because they were subsequently identified as wrong publication types (n=2). This left 48 articles for data extraction and synthesis. To extract the data from the selected studies we constructed a comprehensive tabular form. The principal author conducted initial extraction and all tabular entries were then verified by other co-authors. Inconsistencies were resolved by cross-checking the original reports and reaching consensus through discussion among the authors.

### Data synthesis

Studies were divided in two groups based on different intensities of exposure: 1) occupational exposure, i.e., exposure related to agricultural work, including applying pesticides, and farm activities related to pesticides handling both at home or at work (n=9); and 2) non-occupational exposure, i.e., residential, including para-occupational, exposure after agricultural application, and residential use (n=39).

## RESULTS

### Characteristics of the examined studies

Thirty-one studies had cross-sectional design, 15 were cohort studies (of which one had ambispective design, and two had a mixed design). In 27 studies, exposure was assessed at one time point, while only seven studies evaluated exposure at three or more time points. Neurobehavioural outcomes were also predominantly assessed at a single time point (n=37), while three or more assessments were made in only six studies.

### Assessment of exposure to insecticides

Thirty-nine studies evaluated the effects of non-occupational exposure, such as residential or bystander exposure following agricultural application, pesticide use for treating insects and weeds at home and home gardens and yards, as well as para-occupational (take-home) exposure. The remaining nine studies assessed the effects of occupational exposure related to agricultural work, including applying pesticides as seasonal workers (n=6) or handling pesticides on family farms (n=3).

Twenty-six studies primarily investigated effects of exposure to OP insecticides. Seven studies assessed either combined exposure to OPs and carbamates or non-specified acetylcholinesterase (AChE) inhibitors, while two studies evaluated mixtures that, besides OPs and carbamates, also included pyrethroids (n=1) or pyrethroids and neonicotinoids (n=1). Six studies evaluated the effects of combined OP and pyrethroid exposure, while one study assessed the combination of OPs, pyrethroids, and neonicotinoids. Two studies focused exclusively on pyrethroid exposure. None assessed the effects of exposure to carbamates or neonicotinoids alone. Four studies assessed exposure to mixtures without specifying the classes of insecticides involved.

Regarding exposure assessment methods, 37 studies relied on some form of biomonitoring, including analysis of urinary metabolites (n=23), blood cholinesterase levels (n=17), and pesticide concentration in hair matrices (n=1). Of these, 22 studies relied exclusively on biomonitoring. A smaller proportion of studies (n=5) employed environmental sampling, having analysed pesticide residues in household dust (n=3) or air (n=2), two of which used this analysis as sole exposure indicator.

Questionnaire- or interview-based assessment was the second most common method (n=16), typically involving questions related to occupational exposure, para-occupational (take-home) exposure, maternal and prenatal exposure, residential and environmental exposure, participation in farm activities, and eating crops directly from the field. Three studies relied solely on questionnaire-based exposure assessment.

Eleven studies assessed exposure based on temporal or spatial proximity to agricultural pesticide application, with only two studies using this as the sole indicator of exposure. While 29 studies relied on only one of the aforementioned methods, the rest employed multiple approaches; 17 employed two approaches, and two studies employed three approaches.

Even though studies investigating prenatal exposure were not of interest to us, eleven of our 48 selected studies also assessed residential (n=6) or aggregate (n=5) prenatal exposure.

### Assessment of neurobehaviour

Among the various aspects of neurobehavioural functioning, cognitive functions were assessed most often (n=37), followed by emotional and behavioural functioning (n=15), including internalised and externalised symptoms, and finally sleep (n=2; in one study as an outcome).

Various aspects of cognitive functioning were assessed across the selected studies, and authors were typically interested in examining multiple domains simultaneously. Based on the primary function or process assessed by an instrument used in the study, we identified ten domains of cognitive functioning (Table 1). As instruments often assess related or overlapping constructs, we assigned each to the domain most consistently described as primary in relevant literature (e.g., test manuals or neuropsychological assessment handbooks). For example, although attention and processing speed are closely related, especially as both are usually assessed with timed tasks, tests were categorised based on their scoring focus. There was also a strong conceptual overlap between certain cognitive functions, such as with long-term memory and learning or with working and short-term memory. In the latter, these functions were combined into one domain.

**Table 1:** Overview of assessment tools, study counts, and reported effects across core aspects of neurobehavioural functioning in studies on sleep and neurobehavioural outcomes of insecticide exposure (only adverse and null effect are shown, others are counted)

**Core aspect of neurobehavioural functioning**	**Abbr.**	**Instruments (tests, subtests, tasks, questionnaires)**	**n**			**Adverse effects**			**Null effect**		
			**N**	**O**	**T**	**N**	**O**	**T**	**N**	**O**	**T**
Working memory/immediate (short-term) memory	WM	Digit Span^1^^,^^2^, Match-To-Sample^1^, Letter-Number Sequencing^2^, Picture Span^2^, Arithmetic^2^, Immediate Memory for Faces^3^, Object Memory^4^^,^^14^, Memory for Sentences^5^, Digit String^5^, Spatial Working Memory^6^, Registration^7^, Immediate Memory Span^8^, Children’s Memory Scale, Benton Visual Retention Test, Sternberg working memory task	22	9	31	32 % (7)	78 % (7)	45 % (14)	59 % (13)	22 % (2)	48 % (15)
Long-term memory/learning (includes visual, i.e., non-verbal, and verbal memory)	LTM	Serial Digit Learning^1^, Reverse Learning^1^, Narrative Memory^3^, Delayed Memory for Faces^3^, Object Memory^4^^,^^14^, Copying Recall^5^, Paired Associate Learning^6^, Recall^7^, Delayed Recall^8^, Level of Learning^8^, Children’s Memory Scale, Benton Visual Retention Test, Rey-Osterrieth Complex Figure, Long term memory task^14^	10	6	16	20 % (2)	67 % (4)	38 % (6)	80 % (8)	17 % (1)	56 % (9)
Visuospatial and visuomotor abilities	VSVM	Picture Completion^2^, Block Design^2^^,^^*^^,^^14^, Visual Puzzles^2^, Object Assembly^2^, Design Copying^3^, Geometric Puzzles^3^, Visual motor integration task^4^^,^^14^, Copying^5^, Rey-Osterrieth Complex Figure, Figure drawing^14^, Eye-Hand Coordination^9^	12	6	18	33 % (4)	17 % (1)	28 % (5)	67 % (8)	83 % (5)	72 % (13)
Attention and inhibitory control	AIC	Continuous Performance^1^^,^ ^12^, Selective Attention^1^, Divided Attention^1^, Statue^3^, Auditory Attention & Response Set^3^, Inhibition^3^, Rapid Visual Information Processing^6^, Attention and Calculation^7^, Attention Network Test, Lewis Digit Vigilance Test	9	8	17	56 % (5)	50 % (4)	53 % (9)	33 % (3)	38 % (3)	35 % (6)
(Other) Executive functions	EF	Progressive Ratio^1^, Picture Arrangement^2^, Mazes^2^, Multi-tasking^6^, Orientation^7^, Trail Making Test, Wisconsin Card Sort Test, Behavior Rating Inventory of Executive Function	8	8	16	75 % (6)	75 % (6)	75 % (12)	25 % (2)	25 % (2)	25 % (4)
Sensory and motor function	SMF	Visuomotor Precision^3^, Purdue Pegboard^4^, Santa Ana Form Board^14^, Pegboard^11^^,^^14^, Motor Screening^6^, Motor performance^10^, Name writing^14^, Finger Tapping^1^^,^^14^, Lanthony Desaturated D-15, Gross motor skills and balance^14^	11	7	18	45 % (5)	71 % (5)	56 % (10)	36 % (4)	29 % (2)	33 % (6)
Processing speed/reaction time	PS	Symbol Digit^1^, Simple Reaction Time^1^, Response Reaction^6^, Reaction Time Test^14^, (Digit Symbol) Coding^2^, Symbol Search^2^, Animals^2^, Cancellation^2^	12	9	21	58 % (7)	56 % (5)	57 % (12)	25 % (3)	33 % (3)	29 % (6)
Verbal ability	VA	Information^2^, Similarities^2^, Vocabulary^2^, Comprehension^2^, Comprehension of Instructions^3^, Speeded Naming^3^	14	5	19	50 % (7)	60 % (3)	53 % (10)	43 % (6)	40 % (2)	42 % (8)
Fluid reasoning	FR	Figure Weights^2^, Block Design^2^, Picture Concept^2^, Matrix Reasoning^2^, Series^13^, Classifications^13^, Matrices^13^, Conditions (topology)^13^	6	0	6	50 % (3)	0 % (0)	50 % (3)	50 % (3)	0 % (0)	50 % (3)
General intellectual functioning	GIF	Full scale IQ^2^, Total score^3^^,^^10^, Raven’s Colored Progressive Matrices	14	0	14	57 % (8)	0 % (0)	57 % (8)	43 % (6)	0 % (0)	43 % (6)
Emotional and behavioural functioning	EB	Affect Recognition^3^, Behavior Assessment for Children 2^nd^ edition, Eysenck Personality Questionnaire, Strengths and Difficulties Questionnaire, Mood and Feelings Questionnaire – Child edition, Children’s Depression Inventory 2^nd^ edition, Multidimensional Anxiety Scale for Children 2^nd^ edition, Child Behavior Checklist, Conners’ Rating Scale – Revised Short Version, ADHD Criteria of Diagnostic and Statistical Manual of Mental Disorders 4^th^ edition, Self-Reported Delinquency and Self-Reported Behavior^14^, Parent–child difficulties checklist and parental involvement^14^	14	1	15	57 % (8)	100 % (1)	60 % (9)	14 % (2)	0 % (0)	13 % (2)
Sleep	S	Survey/questionnaire	2	0	2	50 % (1)	0 % (0)	50 % (1)	0 % (0)	0 % (0)	0 % (0)

N – non-occupational exposure studies; O – occupational exposure studies; T – total, all studies;

1 Behavioural Assessment and Research System (BARS);

2 Wechsler Intelligence Scale for Children (WISC) / Wechsler Adult Intelligence Scale (WAIS);

3 Developmental NEuroPSYchological Assessment (NEPSY-II);

4 Pediatric Environmental Neurobehavioural Test Battery (PENTB);

5 Stanford Binet;

6 Cambridge Automated NeuroPsychological Battery (CANTAB);

7 Modified Mini–Mental State Examination for Children (MMMSEC);

8 Children’s Auditory Verbal Learning Test 2^nd^ edition (CAVLT-2);

9 Frostig Developmental Test of Visual Perception 2^nd^ edition (DTVP-2);

10 McCarthy Scale of Children’s Ability (MSCA);

11 Wide Range Assessment of Visual Motor Ability (WRAVMA);

12 Conners’s Kiddie Continuous Performance Test (K-CPT);

13 Culture Fair Intelligence Test (CFIT);

14 Other/not specified, see in original articles;

* also occurs in Fluid reasoning domain, when it was part of an index score from earlier versions of WISC/WAIS

Working memory or immediate (short-term) memory (WM) was the most examined outcome, by 31 studies. Followed processing speed or response time (PS), assessed by 21 studies, and verbal ability (VA) (encompassing verbal comprehension, language, and crystallised intelligence), assessed by 19 studies. Visuospatial and visuomotor abilities (VSVM) and sensory and motor functions (SMF) were each evaluated by 18 studies. Attention and inhibitory control (AIC) were assessed by 17 and other executive functions (EF; such as cognitive flexibility, task-switching, motivation, sequencing, and planning) by 16 studies. Long-term memory or learning (LTM) were assessed by 16 studies. Lastly, general intellectual functioning (GIF) and fluid reasoning (FR) were assessed by 14 and six studies, respectively.

Different editions, subtests, and indices of the Wechsler Intelligence Scale for Children (WISC) and the Wechsler Adult Intelligence Scale (WAIS) (40,41,42,43,44,45,46,47,48,49,50,51,52,53,54,55) were the most common tools used across the selected studies. These were followed by the Behavioural Assessment and Research System (BARS) (46, 56,57,58,59,60,61,62,63) and the Developmental NEuroPSYchological Assessment (NEPSY-II) (64,65,66,67). Among individual tests, the most common were the Trail Making Test (TMT) (40, 41, 46, 51, 57, 60, 62, 63, 68, 69), Benton Visual Retention Test (BVRT) (40, 41, 46, 57, 60, 62, 63), Santa Ana Form Board (PEG-SA) (41, 45, 55, 57, 60, 62, 63), Visual-Motor Integration (VMI) (56, 57, 60, 62), Similarities (57, 60, 62, 63), Block Design (60, 62, 63), and the Wisconsin Card Sorting Test (WCST) (51, 70, 71).

With regard to evaluation of emotional and behavioural functioning, the Child Behavior Checklist (CBCL) was the most common (51, 56, 72,73,74) with answers most often provided by parents (and in some cases teachers). Sleep was evaluated through questionnaire items addressing sleep duration (73) and sleep disorders or trouble sleeping (75). All other instruments were used in no more than one or two studies.

### Confounding factors

Of the 48 selected studies, five did not report controlling for potential confounders in evaluated models, while one study reported adjustment, but did not specify covariates. As the aim of this review was to map which confounders were considered rather than to weight or exclude studies based on this factor, these studies remained in the corpus selected for data synthesis. Most studies that reported controlling for confounders included between six and 12 variables. A few used a smaller set of 3–5 covariates, typically age, sex, ethnicity/race, years of education, maternal education, and the body mass index (BMI). In contrast, some cohort studies evaluated over 15 variables, incorporating psychosocial and biological covariates in addition to standard sociodemographic factors. Studies also differed in strategies used to include confounders. Several studies defined minimal and fully adjusted models a priori, some employed a stepwise modelling approach, while others selected confounders based on statistical thresholds (e.g., p<0.10).

Confounders reported in studies could be categorised in the following groups: 1) demographics and socioeconomic status (e.g., age, sex, maternal education, parental occupation, family income, housing characteristics); 2) health and nutritional status (e.g., BMI, height-for-age, haemoglobin); 3) pesticide exposure adjustments (e.g., creatinine adjustments, timing of sample collection, distance to fields, cohabitation with person applying pesticides); 4) psychosocial and home-environmental factors (e.g., the Home Observation Measurement of the Environment score, Adverse Childhood Experiences score, maternal intellectual functioning, pesticide use at home); and 5) prenatal and lifestyle factors (e.g., maternal smoking and alcohol use, maternal occupational exposure, screen time). In none of those studies was pubertal stage of development controlled for. In only one study (76) concentrations of hormones related to puberty (i.e., testosterone, dehydroepiandrosterone, and oestradiol) were measured in saliva and controlled for in the main analyses.

### Neurobehavioural effects of exposure to insecticides in puberty and adolescence

Overall, 36 studies provided at least some evidence that exposure to insecticides adversely affects neurobehavioural functioning at pubertal or adolescent age. Of them, 28 reported adverse effects on the cognitive functioning, 12 on the emotional and behavioural functioning, and only one on sleep. Five assessed both cognitive and emotional/behavioural functioning (40, 41, 51, 52, 56), but three reported only adverse cognitive outcomes (41, 51, 56).

Eight studies reported no clear evidence of adverse effects or no sufficient data to draw a conclusion. Three studies reported mixed effects (49, 59, 77), while one study reported a positive effect (73).

Table 1 shows the distribution of adverse and null effects across domains by type of exposure (occupational vs. non-occupational). Note that most studies assessed multiple domains at the same time.

#### Working memory or immediate (short-term) memory

Working memory or immediate (short-term) memory, the most commonly assessed outcome (n=31), was reported adversely affected by insecticide exposure by 14 studies, more often in occupational than non-occupational settings, and the effects were predominantly associated with exposure to OPs or OP-containing mixtures (in 13 out of 14 studies).

In most cases, exposure was confirmed by biomarkers, i.e., urinary metabolites and/or blood cholinesterase levels. In some of these studies, the observed effects were also supported by data on work-related exposure or residential proximity to treated areas (40, 41, 53).

One study (78) did not specify the type of exposure (occupational or non-occupational) and relied solely on questionnaire-based data on exposure related to living on farms, including indicators related to work and dietary exposure, and still found evidence of adverse effects. Similarly, the remaining studies reported effects in relation to location (42, 48, 65) or work (62, 63) as exposure proxies. Most of these studies were cross-sectional in design.

#### Processing speed or reaction time

For the next most commonly assessed outcome (n=21), PS, evidence of an adverse effect was reported by 12 studies. In two of them (50, 55), PS was the only affected outcome. Again, the reported adverse effects on PS were mostly associated with exposure to OPs or OP-containing mixtures, in most cases established with the same biomarkers. Four studies found that the adverse effect on PS was associated with residence (42, 47, 48, 58) and one with work with pesticides, especially during the application season (62).

Most of the studies used the cross-sectional design.

#### Verbal ability

Verbal ability was reported adversely affected by exposure to insecticides by 10 out of 19 studies, most of which used the cross-sectional design. Again, the observed adverse effects were associated with exposure to OPs or OP-containing mixtures, except for one study (64), in which exposure involved non-specified insecticides.

Half of these studies used biomarkers as indicators of exposure, supported by information on spatial or temporal proximity to pesticides use, including working with pesticides. The remaining half relied solely on proximity-based proxies, including spatial (42, 47, 65), temporal (64), and occupational (63) exposure proxies. Notably, in three of these studies (47, 63, 65), biomarkers were also analysed but did not appear to drive the reported associations.

#### Sensory/motor function and visuospatial/visuomotor abilities

Adverse effects on SMF were reported by 10 studies, more often in relation to occupational than non-occupational exposure. As for VSVM, null effects were reported by 13 of 18 studies. The observed effects for both outcomes were again primarily related to OPs, OP-containing mixtures, or non-specified insecticides (78).

The five studies that reported adverse insecticide effects on VSMS (40, 41, 52, 64, 67) relied on biomarkers and employed cross-sectional or mixed (64) design.

Of the ten studies that reported adverse effects on SMF, five primarily relied on biomarkers (41, 52, 57, 60, 69) and the other five on proximity-based proxies, i.e. spatial (56, 58), temporal (67), and/or work-related (62, 78). Six studies employed a cross-sectional design, while the remaining four were longitudinal and compared adolescents exposed either occupationally (57, 60, 62) or para-occupationally (56) to a low exposure group (control).

Notably, two studies reported improved sensory and motor function. The cross-sectional pilot study conducted in Argentina by Martos Mula et al. (49) compared two groups of children aged 7–10 years: one residing in an agricultural town with a high risk of exposure, and the other in a livestock-farming town with a lower exposure risk. The authors found that children in the high-risk group made fewer errors on task assessing gross motor skills. Similarly, Fiedler et al. (59) compared two small cohorts of children aged 6–8 years in Thailand: one group living on rice farms and the other on aquaculture farms. While higher levels of exposure to pyrethroids predicted poorer cognitive performance for WM and PS, greater exposure to OPs was associated with improved SMF and AIC.

#### Attention and inhibitory control

Of the 17 studies assessing AIC, adverse effects were reported by nine studies and were primarily associated with OPs, OP-containing mixtures, or mixtures containing non-specified insecticides (58, 64). These effects were observed in relation to residential (48, 56, 58, 65), seasonal (64, 67), and/or work-related (48, 58, 60, 62) exposure, and/or higher OP metabolite levels or reduced AChE/BChE blood activity (60, 66).

Five studies employed a cross-sectional design, three were longitudinal, and one utilised a mixed design.

#### Executive functions

EFs were the most affected outcome, with adverse effects reported by 12 of 16 studies, primarily in association with exposure to OPs, OP-containing mixtures, or mixtures containing non-specified insecticides (78). Two studies (46, 51), in fact, found only the adverse effects on EFs, while one study (49) also reported mixed findings for other outcomes.

Although adverse effects were most often associated with exposure that was confirmed with biomarkers, including urinary metabolites, OP concentrations in hair samples, and/or blood cholinesterase levels, some studies also reported correlations with other indicators, such as occupational (40, 60) or para-occupational (41) exposure. In a few studies, however, adverse effects were observed only with reported activities on a farm (58, 62, 78) or living in an agricultural area (49), but not with biomarkers.

Nine studies had cross-sectional design, three were cohort studies.

#### Long-term memory or learning

LTM was assessed by 16 studies, but only six reported adverse effects, with evidence more often related to occupational than non-occupational exposure. Associations were primarily observed with exposure to OPs (57, 62, 63), OP-containing mixtures (65, 66), or non-specified insecticides (78).

One study (58) comparing 10–18-year-olds from a rural farming community, who alternated weekly schedule between school and farm work, with urban children found that rural children outperformed urban in LTM. The study did not specify to which insecticides the rural children were potentially exposed nor were they objectively measured in this study.

Studies that reported evidence of adverse effects typically used objective measures, such as biomarkers. With the exception of one study (66), the observed effects were predominantly associated with farm activities (57, 62, 63, 78) or living in close proximity to crops (65).

Four of these studies were cross-sectional and two cohort (57, 62).

#### Fluid reasoning

FR was assessed using the WISC in five cross-sectional and one ambispective cohort study investigating non-occupational exposure to OPs or OP-containing mixtures. Adverse effects were reported by three studies and were associated with OP exposure: in the first (44), exposure was evidenced by elevated urinary OP metabolite levels during periods of intensive pesticide use, in the second (53) by elevated urinary OP metabolite levels in children living in rural areas, and in the third by residential proximity to a greenhouse (47).

#### General intellectual functioning

GIF was assessed by 14 studies of varying designs (mostly cross-sectional) that investigated the effects of non-occupational aggregate and/or residential exposure to OPs or mixtures. Eight reported adverse effects, which were associated with cholinesterase inhibition (66, 79, 80), higher urinary OP metabolite levels (44, 53), and/or temporal (44, 67) or spatial (47, 53, 65, 67, 80) proximity to heightened pesticide use.

**Table 2:** Summary of studies investigating sleep and neurobehavioural effect of exposure to insecticides

	**Ref.**	**Study design**	**Country, region, cohort, and start year**	**Sample size (% of boys)**	**Groups**	**Age range (years)**	**Type of insecticide**	**Exposure assessment**	**Neurobehavioural assessment**	**Main findings**
Non-occupational (residential) exposure studies	(82)	CO	China, Asia SMBCS 2009–2010	n=1266 mother-child pairs (54 %)	Formed *a posteriori* 1. Continuously low (n=197) 2. Early high (n=286) 3. Mid-term high (n=104)	1–10 (within subject)	OP (chlorpyrifos, chlorpyrifos-methyl) and CARB (carbofuran)	urinary OP (TCPy) and CARB (CFP) metabolites	SDQ, Parent/caregiver assessment ADHD Criteria of Diagnostic and Statistical Manual of Mental Disorders, Fourth Edition (ADHD-DSM-IV) rating scale	Evidence of adverse effects on EB functioning in children with exposure during infancy and toddlerhood (↑OP metabolites)
	(65)	CS	Ecuador, South America ESPINA 2008	n=313 (51 %)		4–9	mixture, including OP, NEO, and PYR	residential proximity to crops (ArcGIS)	NEPSY-II (AIC, L, ML, VP)	Evidence of adverse effects on WM, LTM, AIC, VA, and GIF related to living in close proximity to greenhouse floricultural crops
	(67)	CS	Ecuador, South America ESPINA 2008	n=313 (51 %)		4–9	OP, CARB	AChE blood lev. (E); temporal proximity to heightened pesticide use; residential proximity to plantation (ArcGIS)	NEPSY-II (AIC, L, ML, VP, SM)	Evidence of adverse effects on VSVM, AIC, SMF, and GIF related to peak pesticide spray seasons in agricultural communities
	(66)	CS	Ecuador, South America ESPINA 2008	n=313 (52 %)		4–9	OP/CARB	AChE blood levels (E)	NEPSY-II (AIC, L, ML, VP, SM)	Evidence of adverse effects on LTM, AIC, and GIF related to ↓AChE activity in boys
	(61)	CS-pilot	Costa Rica, North America 2007	n=35 (NR)	1. Children of farm owners in Las Mellizas, conventional farming (n=18) 2. Children of workers in La Amistad, organic coffee plantations (n=17)	4–10	OP, PYR	urinary OP (PNP, IMPy, TCPy) & PYR (3-PBA) metabolites	BARS (DST, TAP, MTS, CPT, DAT), Figure drawing, Long term memory test	Conclusion could not be drawn based on reported data
	(64)	mixed	Ecuador, South America ESPINA 2008	n=309 (50 %)		T1 (2008): 4–9 years; T2&3 (2016): 11–17 years	mixture, types not specified	AChE blood levels (E); questionnaire; residential proximity to crops (ArcGIS); temporal proximity to heightened pesticide use	NEPSY-II (AIC, L, ML, VP, SP)	Evidence of adverse effects on VSVM, AIC, and VA lasting up to 90+ days after peak pesticide spray seasons
	(55)	CS-pilot	Ecuador, South America	n=79 mother-child dyads (47 %)	Formed *a posteriori* 1. Prenatally exposed (n=37) 2. Controls (n=35; no prenatal pesticide exposure)	5–8	OP	AChE blood levels (E); urinary OP metabolites (DAPs); interview	SRT, PEG-SA, DST (WISC-IV), Copying test (Stanford-Binet), FTT	Prenatal exposure important risk factor. Current OP exposure (↑OP metabolites) only predicted poorer PS
	(51)	CO	USA (Arizona), North America CPS 1998	n=48 (46 %)	1. Exposed before (n=25) 2. Non-exposed before (n=23)	5–9	OP	urinary OP metabolites (DAPs)	WISC-III SF, CMS, WCST, TMT; CBCL/4–18, TRF	No strong evidence of adverse effects. Current OP exposure (↑OP metabolites) only predicted poorer EF
	(72)	CS	USA (Pacific Northwest), North America 2008	n=215 parent-child pairs (54 %)	1. Agricultural families (n=122) 2. Non-agricultural (n=50)	5–12	OP	pesticide concentrations (house dust samples)	CBCL/6–18	Evidence of adverse effects on EB related to OP concentration in house dust
	(56)	CO	USA (Pacific Nortwest), North America 2008	n=328 (55 %)	1. Agricultural group (n=155) 2. Non-agricultural (n=60)	5–12	OP	OP residues (home carpet dust); questionnaire	BARS (DST, TAP, MTS, SDT, CPT, DAT), OMT, PEG-P, VMI, Name writing; CBCL	Evidence of adverse effects on AIC and SMF related to OP exposure in children from agricultural families. Mixed effects on WM
	(53)	CS	Mexico, North America 2010	n=105 (51 %)		5–14	OP	urinary OP metabolites (DAPs)	WISC-IV (VIC, PRI, WMI, PSI, FSIQ)	Evidence of adverse effects on WM, PS, VA, FR, and GIF related to OP exposure (↑OP metabolites) in children living in rural area
	(59)	CO	Thailand, Asia	n=54 (58 %)	1. Rice farming (n=24) 2. Aquaculture farming (n=29)	6–8	OP, PYR	urinary OP (DAPs, TCPy) & PYR (3-PBA, DCCA) metabolites	(TAP, DAT, DST, SDT, MTS, CPT), PENTB (PEG-P, VMI, OMT)	Mixed effects related to OP and PYR exposure during high and low pesticide use season in aqua- and agriculture communities. ↑PYR metabolites predicted poorer PS and WM performance in certain instances, while ↑OP metabolites predicted improvement in AIC and SMF
	(45)	CS	Ecuador, South America	n=87 mother-child dyads (58 %)	*Formed a posteriori* 1. Prenatal maternal exposure (n=35) 2. Prenatal paternal exposure (n=23) 3. Controls (n=26; no prenatal pesticide exposure)	6–8	OP	AChE blood levels (E); OP urinary metabolites (DAPs); interview	FTT, PEG-SA, K-CPT, Stanford-Binet (Copying, Copying Recall, Memory for Sentences, Digit String), DST (WISC-IV), RCPM	Prenatal exposure more harmful than current exposure. No evidence of adverse effects of current exposure
	(52)	CS	Costa Rica, North America 2007	n=190 (49 %)	1. Large plantains using pesticides 2. Small plantains using pesticides 3. Small organic plantains	6–9	OP, PYR	urinary OP (TCPy) & pyrethroids (3-PBA) metabolites	WISC-IV (Block Design, Picture Concepts, Matrix Reasoning, DST, Letter Number Sequencing, Coding, Symbol Search, Cancellation); LDD-15, ROCF Copy and Delayed recall trials, CAVLT-2 (Immediate Memory Span, Delayed Recall, Level of Learning), DTVP-2, WRAVMA, RRT; CPRS-R	Evidence of adverse effects on WM (in boys), VSVM, SMF, PS (in girls), and EB functioning related to OP and PYR exposure (↑OP & PYR metabolites) in children living near plantations
	(47)	CS	Iran, Asia 2019	n=128 (45 %)		6–9	OP	OP concentrations (ambient air samples; within a 50-meter radius from the greenhouse); residential proximity to the greenhouse (GPS)	WISC-IV (VIC, PRI, WMI, PSI, FSIQ)	Evidence of adverse effects on PS, VA, FR, and GIF related to greenhouse density
	(83)	CS	Canada, North America CHMS, cycle 1 2007	n=1081 (51 %)		6–11	OP, PYR	urinary OP (DAPs) & PYR metabolites (4-F-3-PBA, cis-DBCA, cis-DCCA, trans-DCCA, trans-DCCA)	SDQ	Evidence of adverse effects on EB functioning related to pesticide exposure (↑PYR metabolites; pesticide used to treat pets/head lice)
	(73)	CO	UK, France, Spain, Lithuania, Norway, Greece, Europe HELIX study: BiB, EDEN, INMA, KANC, MoBa, Rhea 2013	n=1301 mother-child dyads (55 %)		6–11	OP	urinary OP metabolites (DAPs)	CBCL/6–18, CPRS-R; questionnaire items (sleep duration)	Adverse effect on EB functioning related to prenatal exposure. Childhood exposure (↑OP metabolites) predicted better EB functioning
	(85)	CS	UK, France, Spain, Lithuania, Norway, Greece, Europe HELIX study: BiB, EDEN, INMA, KANC, MoBa, Rhea 2013	n=1301 mother-child pairs (55 %)		6–11	OP	urinary OP metabolites (DAPs)	ANT and other tests (not described)	No evidence of adverse effects
	(50)	CS-pilot	Chile, South America	n=25 (56 %)	1. Near agricultural land, Maule (n=15) 2. Not near agricultural land, Talca (n=10)	6–11	OP	urinary OP metabolites (DAPs) levels; school location (urban or rural)	WISC-III (VCI, POI, FDI, PSI, FSIQ)	No strong evidence of adverse effects. ↑OP metabolite levels only predicted poorer PS
	(44)	A	Spain, Europe 2009	n=315 mother-child dyads (51 %)		6–11	OP	urinary OP metabolites (DAPs); questionnaire; pre- and postnatal residential proximity to crops	WISC-IV (VIC, PRI, WMI, PSI, FSIQ)	Evidence of adverse effects on GIF, VA, PS and FR related to high pesticide use season and OP exposure (↑OP metabolites), stronger in males
	(69)	CS	Malaysia, Asia	n=683 (50 %)	1. 7–8.5-year-olds (n=339) 2. 10–11-year-olds (n=344)	6–11	OP	ChE blood levels	WHO-NCTB (TMT), MSCA (motor scale)	Evidence of adverse effects on SMF and EF related to ↓blood ChE levels
	(68)	CS	Iran, Asia 2021	n=114 (50 %)	1. Exposed (agricultural regions; n=57) 2. Controls (urban areas; n=57)	6–13	OP	pesticides concentration (hair samples)	TMT A & B	Evidence of adverse effects on EF related to OP concentration in hair, stronger in females
	(80)	CS	Malaysia, Asia	n=389 (20 %)	1. Children that live <100 m from paddy fields (n=227) 2. Children that live >1000 m from the agricultural site (n=162)	7–8.5	OP	ChE blood levels; residential proximity to paddy fields	MSCA	Evidence of adverse effects on GIF related to ↓ ChE levels in children living in close proximity to agricultural fields
	(42)	CS	Columbia, South America 2019	n=232 parent-child dyads (50 %)		7–10	mixture, containing primarily OP, PYR, fungicides	questionnaire; info from stores about most bought pesticides in the area	WISC-IV (VIC, PRI, WMI, PSI, FSIQ)	Evidence of adverse effects on WM, VA, and PS (in girls) related to reported exposures at home or school
	(118)	CS	Lebanon, Asia 2017	n=464 parent-child dyads (46 %)		7.5–13	mixture, types not specified	questionnaire	Cattell 2 test (Series, Classification-differences, Matrices, Conditions)	No adverse effect
	(43)	CS	USA (North Carolina), North America PACE5 2018–2019	n=141 (50 %)	1. Farmworking (n=76) 2. Non-farmworking (n=65)	8	OP, PYR	passive pesticide exposure (silicone wristbands); questionnaires	WISC-V (VCI, VSI, FRI, WMI, PSI, FSIQ)	No evidence of adverse effects, except mixed effects observed on VA
	(79)	CS	Indonesia, Asia	n=56 (45 %)		9–11	OP	AChE blood levels (E); questionnaire	MMMSEC (orientation, attention, registration, recall, and language)	Evidence of adverse effects on WM, EF, and GIF related to ↓AChE activity
	(41)	CS	Egypt, Africa 2017	n=150 (0 %)	1. Exposed (n=100) 2. Controls (n=50)	9–18	primarily OP (chlorpyrifos)	AChE blood levels (S); questionnaire	WAIS (Information, Similarities, Arithmetic, Block Design, Digit Symbol, DST), BVRT, TMT, Santa A dexterity test, Beery Visual Motor Integration Test; questionnaire	Evidence of adverse effects on WM, VSVM, EF, SMF, and VA in girls living in agricultural areas, related to para-occupational exposure, and ↓AChE levels
	(49)	CS-pilot	Argentina, South America	n=71 (49 %)	1. High risk of exposure, Yuto (n=42) 2. Low risk of exposure, León (n=29)	7–10	OP, CARB	BChE (P) and AChE (E) blood levels; residential location (agricultural or livestock-farming area)	WISC-III (DST, Digit-Symbol, Mazes), gross motor skills and balance tasks	Mixed effects. Evidence of adverse effect on EF, and positive effect on SMF related to residential exposure to pesticides. Null effect on WM and PS
	(81)	mixed	Ecuador, South America ESPINA 2008	n=545 (49 %)		11–17	OP/CARB	AChE blood levels (E)	CDI-2, MASC-2 Child self-report	Evidence of adverse effects on EB functioning related to ↓AChE activity, especially in female and younger adolescents
	(84)	CS	USA (Oklahoma), North America 2013	n=91 parent-youth dyads (60 %)		M = 14.4 (1.7)	PYR	pesticide concentration (wipe samples from home)	Mood and Feelings Questionnaire, Child version (MFQ-C); Parent–child difficulties checklist; Parental involvement (7 items)	Evidence of adverse effects on EB functioning in adolescents related to PYR concentration in house dust
	(76)	CO	Ecuador, South America ESPINA 2016	n=554 (49 %)		12–17	OP/CARB	AChE blood levels (E)	CDI-2, MASC-2 Child self-report	Evidence of adverse effects on EB functioning related to ↓AChE activity, stronger in females and older adolescents
	(115)	CO	USA (California), North America CHAMACOS 1999–2000	n=537 mother-child dyads (46 %)		14, 16, 18 (within subject)	OP	maternal and child urinary OP metabolites (DAPs)	BASC-2: Parent form & Self-Report of Personality	Evidence of adverse effects related to prenatal, but not childhood, OP exposure
	(74)	CS	Spain, Europe INMA-Granada 2017	n=151 (100 %)		15–17	OP, PYR, CARB	urinary OP (TCPy, IMPy, MDA, DETP, DEDTP), PYR (3-PBA, DCCA) and CARB (1-N) metabolites	CBCL/6–18	Evidence of adverse effects on EB functioning related to exposure to a combination of insecticides (↑OP & PYR metabolites)
	(54)	CO	USA (California), North America CHAMACOS 1999–2000	n=534 mother-child dyads (46 %)		16–17	OP	maternal and child urinary OP metabolites (DAPs)	WISC-IV (FSIQ), BRIEF; Self-Reported Delinquency and Self-Reported Behavior scales, Conner’s ADHD Index	Limited evidence of adverse effects on EB functioning related to prenatal OP exposure only.
	(116)	CO	USA (California), North America CHAMACOS 1999–2000	n=527 mother-child dyads & additional 305 dyads (in 2009–2011) (49 %)		16 & 18 (within subject))	neurotoxic pesticides, including OP, PYR, NEO, CARB	residential proximity to agricultural pesticide use (within 1 km from home)	BASC-2 Parent version, BASC-2 Self-Report of Personality	Mixed evidence of adverse effects on EB functioning related to living in close proximity to agricultural pesticide use (OP, PYR, CA, NE) during both prenatal and early childhood (0–5 y) development
	(75)	CS	USA (national, representative), North America NHANES 2007	n=17551 (899) (52 %)		16–20	PYR	urinary PYR metabolites (3-PBA, Trans-DCCA, 4-F-3-PBA, Cis-DBCA)	Questionnaire items (sleep disorder, trouble sleeping)	Evidence of adverse effects on sleep related to PYR exposure (↑ PYR metabolites) in males
	(70)	CO	USA (California), North America CHAMACOS 1999–2000	n=537 mother-child dyads (46 %)		18–19	OP	OP metabolites (DAPs)	WCST, Sternberg working memory test, Pyramids and Palm Trees Task	No evidence of adverse effect related to prenatal and childhood exposure. In boys, childhood OP exposure (↑OP metabolites) only predicted worse WM
	(71)	CO	USA (California), North America CHAMACOS 1999–2000	n=537 mother-child dyads (46 %)		18–19	OP	maternal and child urinary OP metabolites (DAPs)	WCST; Sternberg working memory task; Pyramids and Palm Trees	No strong evidence of adverse effects related to prenatal or childhood OP exposure
	(48)	CS	Argentina, South America	n=87 (NR)	1. High exposure, Monterrico (n=31) 2. Medium exposure, Perico (n=33) 3. Low exposure, San Salvador de Jujuy (n=23)	11–16	OP, CARB	BChE (P) and AChE (E) blood levels; residential location (rural, in close proximity to plantations, or urban)	WISC (DST, Digit-Symbol), Lewis Digit Vigilance Test	Evidence of adverse effects on WM, AIC, and PS (in moderate exposure group) related to residential exposure to pesticides
	(78)	CS	South Africa, Africa CapSA 2017	n=1001 (47 %)		9–16	mixture, types not specified	questionnaire	CANTAB (MS, RR, SWM, PAL, MTT, RVP)	Evidence of adverse effects on WM, LTM, EF, and SMF in children living on farms, related to eating crops and engaging in farm-related activities. Mixed effects on AIC
	(40)	CS	Egypt, Africa 2005	n=100 (100 %)	1. Younger applicators (n=30) 2. Older applicators (n=20) 3. Younger controls (n=30) 4. Older controls (n=20)	9–18	primarily OP (chlorpyrifos)	AChE blood levels (S/P); questionnaire	WAIS (Information, Similarities, Arithmetic, Block Design, Digit Symbol, DST), BVRT, TMT; EPQ	Evidence of adverse effects on WM, VSVM, EF PS, VA, and EB related to pesticide application and ↓ AChE levels
	(58)	CS	Brazil, South America 2001	n=66 (55 %)	1. Rural residents (n=38) 2. Urban residents (n=28)	10–18	mixture, types not specified	questionnaire; residential location (rural or urban)	BARS (CPT, MTS, DST, PRT, SRT, SAT, SDL, SDT, TAP)	Evidence of adverse effects on AIC, EF, SMF, and PS related to farming activities in younger adolescents. In whole sample, rural residents performed better on LTM than urban
	(57)	CO	Egypt, Africa 2016	n=242 (100 %)	1. Applicators (n=177) 2. Non-applicator (n=65)	12–18	OP (chlorpyrifos)	urinary OP (TCPy) metabolites over 1 year	BARS (MTS, CPT, DST, SDT, SDL, TAP, SRT), Similarities, BVRT, TMT-A & -B, VMI, PEG-SA	Evidence of adverse effects on WM, LTM, and SMF related to pesticide application (↑OP metabolites)
	(46)	CS	Egypt, Africa 2005 & 2009	n=120 (100 %)	1. Applicators 2005 cohort (n=41) 2. Applicators 2009 cohort (n=21) 3. Non-applicators 2005 cohort (n=38) 4. Non-applicators 2009 cohort (n=20)	12–18	OP (primarily chlorpyrifos)	BChE blood levels (S/WB); questionnaire	BARS, BVRT, WAIS-R, TMT; Neurological assessment	No strong evidence of adverse effects. ↓ BChE activity only predicted worse performance on EF.
	(63)	CS	Egypt, Africa 2009	n=41 (100 %? NR)	1. Applicators (n=21) 2. Non-applicators (n=20)	12–18	OP (primarily chlorpyrifos)	AChE (E) and BChE (P) blood levels; urinary OP metabolite (TCPy); questionnaire	BARS (MTS, SDL, DST, CPT, SAT, TAP, SRT, SDT), BVRT, PEG-SA, TMT, Similarities, Block Design	Evidence of adverse effects on WM, LTM, and VA in adolescent pesticide applicators
	(60)	CO	Egypt, Africa 2010	n=84 (100 %? NR)	1. Applicators (n=46) 2. Non-applicators (n=38)	12–21	OP (primarily chlorpyrifos)	AChE and BChE blood levels; urinary OP metabolite (TCPy)	BARS (CPT, DST, SDT, TAP), PEG-SA, VMI, TMT, BVRT, Similarities, Block Design	Evidence of adverse effects on WM, AIC, EF, SMF, PS, VA in adolescent pesticide applicators related to ↑OP metabolites and ↓AChE and BChE activity
	(62)	CO	Egypt, Africa 2010	n=98 (100 %)	Formed *a posteriori* (TCPy concentrations): 1. High exposure (n=47) 2. Low exposure (n=42)	12–21	OP (primarily chlorpyrifos)	urinary OP metabolite (TCPy); questionnaire	BARS (TAP, SAT, DST, SDL, SDT, SRT, RLT), BVRT, TMT, PEG-SA, Similarities, Block Design, VMI	Evidence of adverse effects on WM, LTM, AIC, EF, SMF, and PS in adolescents highly exposed to OP pesticides, especially during the application season

**Table 2 explanation** 1-N – 1-Naphthol; 3-PBA – 3-phenoxybenzoic acid; 4-F-3-PBA – 4-fluoro-3-phenoxybenzoic acid; A – ambispective study; AChE – acetylcholinesterase; ADHD – attention-deficit/hyperactivity disorder; ADHD-DSM-IV – ADHD Criteria of Diagnostic and Statistical Manual of Mental Disorders IV rating scale; AIC – attention & inhibitory control; ANT – Attention Network Test; BARS – Behavioural Assessment and Research System; BASC – Behaviour Assessment System for Children; BChE – butyrylcholinesterase; BRIEF – Behaviour Rating Inventory of Executive Function; BVRT – Benton Visual Retention Test; CANTAB – Cambridge Automated NeuroPsychological Battery; CARB – carbamate; CAVLT-2 – Children’s Auditory Verbal Learning Test; CBCL – Child Behavior Checklist; CDI-2 – Children’s Depression Inventory; CFIT – Culture Fair Intelligence Test; CFP – carbofuran phenol; ChE – plasma cholinesterase; CMS – Children’s Memory Scale; CO – cohort (longitudinal) study design; CPRS-R – Conners’ Parent Rating Scale-Revised; CPT – Continuous Performance Test; CS – cross-sectional study design; DAPs – dialkyl phosphate metabolites; DAT – Divided Attention Test; DBCA – dibromochloropropane acid; DCCA – cis-/trans-3-(2,2-dichlorovinyl)-2,2-dimethylcyclopropane carboxylic acid; DEDTP – diethyldithiophosphate; DETP – diethylthiophosphate; DST – Digit Span Test; DTVP-2 – Frostig Developmental Test of Visual Perception; E – erythrocyte; EB – emotional & behavioural functioning; EF – executive functions (other); EPQ – Eysenck Personality Questionnaire; FDI – Freedom from Distractibility Index; FR – fluid reasoning; FRI – Fluid Reasoning Index; FSIQ – Full Scale Intelligence Quotient; FTT – Finger Tapping Test; GIF – general intellectual functioning; IMPy – imidacloprid pyridine; K-CPT – Conners’s Kiddie Continuous Performance Test; L – language domain; LDD-15 – Lanthony Desaturated D-15; LTM – long-term memory/learning; MASC-2 – Multidimensional Anxiety Scale for Children; MDA – malathion dicarboxylic acid; MFQ-C – Mood and Feelings Questionnaire – Child version; ML – memory and learning domain; MMMSEC – Modified Mini – Mental State Examination for Children; MS – motor screening; MSCA – McCarthy Scale of Children’s Ability; MTS – Match-To-Sample; MTT – Multi-Tasking Test; NEO – neonicotinoid; NEPSY-II – Developmental NEuroPSYchological Assessment; OMT – Object Memory Test; OP – organophosphate; P – plasma; PAL – Paired Associates Learning; PEG-P – Purdue Pegboard; PEG-SA – Santa Ana Form Board; PENTB – Paediatric Environmental Neurobehavioural Test Battery; PNP – para-nitrophenol; POI – Perceptual Organisation Index; PRI – Perceptual Reasoning Index; PRT – Progressive Ratio Test; PS – processing speed/reaction time; PSI – Processing Speed Index; PYR – pyrethroid; RCPM – Raven’s Coloured Progressive Matrices; RLT – Reversal Learning Test; ROCF – Rey-Osterrieth Complex Figure; RR – response reaction; RRT – Rapid Response Task; RVP – Rapid Visual Information Processing; S – serum; SAT – Selective Attention Test; SDL – Serial Digit Learning; SDQ – Strengths and Difficulties Questionnaire; SDT – Symbol Digit Test; SM – Sensorimotor domain; SMF – sensory & motor function; SP – Social Perception domain; SRT – Simple Reaction Time; SWM – Spatial Working Memory; TAP – tapping; TCPy – 3,5,6-trichloro-2-pyridinol; TMT – Trail Making Test; VA – verbal ability; VCI – Verbal Comprehension Index; VMI – visual motor integration; VP – visuospatial processing domain; VSI – Visuospatial Index; VSVM – visuospatial and visuomotor abilities; WAIS – Wechsler Adult Intelligence Scale; WB – whole blood; WCST – Wisconsin Card Sorting Test; WHO-NCTB – WHO Neurobehavioural Core Test Battery; WISC – Wechsler Intelligence Scale for Children; WM – working memory/short-term (immediate) memory; WMI – Working Memory Index; WRAVMA – Wide Range Assessment of Visual Motor Ability

#### Emotional or behavioural functioning

Emotional or behavioural functioning (EB) was assessed by 15 studies, 14 of which investigated non-occupational exposure. Nine reported adverse effects, including symptoms of depression, hyperactivity/inattention, conduct issues, and somatic complaints. All these studies objectively confirmed exposure, either with low blood cholinesterase levels (40, 76, 81), high urine OP (82) and/or pyrethroid metabolite levels (52, 74, 83), or high OP (72) or pyrethroid (84) residues in house dust.

One large cohort (HELIX) study (73) with 1301 mother child-pairs found some unexpected associations between a wide range of prenatal and childhood environmental exposures, including those to OP insecticides, and emotional and behavioural problems. While prenatal exposure was associated with prominent externalising symptoms, childhood exposure was associated with milder externalising symptoms and ADHD scores in children aged 6 to 11 years. Sleep was also assessed in this study, not as an outcome of exposure but as a lifestyle factor, with longer sleep duration showing a beneficial effect on behavioural problems in this age group.

#### Sleep

Finally, sleep as an outcome was assessed by only one large cross-sectional (NHANES) study on a nationally representative sample from the US (75). The authors examined the association between pyrethroid exposure, measured via urinary concentrations of the 3-phenoxybenzoic acid (3-PBA) metabolite, and self-reported sleep problems in male and female adolescents aged 16–20 years. The study found a significant positive association in males but not in females.

### Differences between occupational and non-occupational exposure to insecticides

Studies of occupational exposure were mostly conducted in Africa (n=7) and South America (n=2), whereas non-occupational exposure (i.e. everyday-life) was mostly investigated in North America (n=15), South America (n=11), Asia (n=8), Europe (n=4), and only one in Africa.

Exposure related to agricultural work (i.e., handling pesticides) was mostly investigated in relatively small samples, typically ranging from 60 to 120 participants. The smallest sample consisted of 41 participants (63) and the largest of 1,001 participants (78).

Researchers typically compared a higher exposure group (e.g., pesticide applicators or children living on agricultural farms or in a rural area) to a lower exposure group (e.g., non-applicators or children living on aquaculture farms or in an urban setting). Some studies compared more than two groups. One included three groups with different pesticide exposure (48), one included subgroups of two different cohorts (60), and another subgroups of applicators and non-applicators further divided by age (younger vs older) (40).

Most of the studies were conducted on male populations only, as female adolescents were generally not employed in pesticide application or related agricultural work (57). While four studies explicitly reported that participants were male adolescents (40, 46, 57, 62), three other studies did not specify gender but were most likely also limited to male populations (60, 63), while in one (48) gender could not be determined from the available data. In this study the authors compared three exposure groups based on geographic proximity to agricultural activity and involvement in rural activities. In the remaining two studies (58, 78), the proportion of male and female participants was approximately equal.

The age of participants across the studies ranged from 9 to 21 years. Studies on occupational exposure were primarily interested in cognitive functions, but one also evaluated emotional and behavioural functioning (40) and one included neurological assessment (46). Adverse cognitive effects involved working memory/immediate (short-term) memory (78 %), executive functions (75 %), sensory and motor functions (71 %), long-term memory/learning (67 %), verbal ability (60 %), and processing speed/reaction time (56 %).

In studies exploring non-occupational residential exposure, sample sizes varied greatly, from as few as 25 participants in a small pilot study (50) to over 1,000 in large-scale studies (73, 82, 83, 85). Most studies, however, clustered between sample sizes of 300 and 600 (n=14).

The age of participants also ranged widely, with most studies focusing on children between 6 and 18 years, but their median values of the lower and upper age limits were 7 and 12.5 years, respectively.

Most studies included a matching number of male and female participants, with the proportion of boys varying between 45 and 60 %. One study (61) did not specify participants’ gender. One study (41) included only girls, while the other (80) included boys as well, but they were underrepresented. One study (74) included boys only.

Adverse effects were most often observed for executive functions (75 %), processing speed/reaction time (58 %), general intellectual functioning (57 %), emotional and behavioural functioning (57 %), attention and inhibitory control (56 %), verbal ability (50 %), and fluid reasoning (50 %).

### Findings from European studies

Among the selected studies, four (44, 73, 74, 85) were conducted in Europe, all investigating non-occupational exposure. Two of them (73, 85) were part of the HELIX project, which is a multicentre European cohort study that integrates data from six population-based birth studies to investigate the effects of early-life environmental chemical, physical, and social exposures on child health and development, namely the Born in Bradford study (BiB, UK), the Early Pre- and Postnatal Determinants of Psychomotor Development and Health of the Child study (EDEN, France), the Environment and Childhood Project (INMA, Spain), the Kaunas Cohort (KANC, Lithuania), the Norwegian Mother, Father and Child Cohort Study (MoBa, Norway), and the Rhea Mother-Child Cohort study (Rhea, Greece). Two other studies included in this review (44, 74) were based in Granada, Spain, but one of them (74) overlaps with the INMA cohort included in the HELIX project.

Although all four studies investigated the neurodevelopmental effects of early-life exposure to non-persistent pesticides (among other chemicals), they varied in design, analytical approach, exposure assessment methods, and neurobehavioural outcomes. Also, three of the four studies focused on school-age girls and boys between the ages of 6 and 11 years, whereas one (74) focused on male adolescents aged 15 to 17 years.

This cross-sectional study in boys (74) examined associations of urinary metabolites of OPs, carbamates, and pyrethroids with emotional and behavioural problems and with the brain-derived neurotrophic factor (BDNF) levels as exposure effect biomarker, measured at two levels of biological complexity (DNA methylation and serum protein levels). Higher urinary levels of OP metabolites [2-isopropyl-4-methyl-6-hydroxypyrimidine (IMPy) and 3,5,6-trichloro-2-pyridinol (TCPy)] were associated with increased behavioural problems, including total, thought, social, and externalising problems, as well as more specific problems such as rule-breaking and aggressive behaviour, as assessed by the CBCL/6–18. Models of mixture effects identified specific metabolites of OPs [IMPy and malathion diacid (MDA)], pyrethroids [dimethylcyclopropane carboxylic acid (DCCA)], and of the ethylene-bis-dithiocarbamate fungicides [ethylene thiourea (ETU)] as the strongest contributors to adverse behavioural outcomes, particularly with withdrawn and social problems. Additionally, several OP [IMPy, MDA, and diethyl thiophosphate (DETP)] and carbamate (1-naphthol) metabolites were linked to reduced serum BDNF levels, while MDA, pyrethroid metabolite 3-phenoxybenzoic acid (3-PBA), and ETU were associated with increased DNA methylation at specific CpG sites. Furthermore, intermediate and high levels of BDNF protein were associated with fewer thought and rule-breaking symptoms, while methylation changes at specific CpG sites were linked to both increases (CpG6) and decreases (CpG2) in various internalising and attention problems.

In an ambispective cohort study González-Alzaga et al. (44) found that higher postnatal OP exposure, assessed via urinary OP metabolite levels as well as temporal and residential proximity to areas of agricultural pesticide use was consistently associated with poorer general intellectual functioning and verbal comprehension, with sex-specific patterns emerging across different exposure indicators. Additional deficits were observed in fluid reasoning and processing speed, with the latter also linked to higher prenatal exposure indices in boys.

Maitre et al. (73) and Fabbri et al. (85) analysed data from the HELIX project. The former group investigated insecticide effects on emotional and behavioural functioning and the latter on cognitive functioning. Maitre et al. (73) used comprehensive and systematic approach to evaluate a wide range of exposures (n=88), including insecticides, using exposome-wide association models that were adjusted for co-exposures to identify the most relevant environmental contributors. The results of this study have been described in the *Emotional or behavioural functioning* subsection. In their cross-sectional study, Fabbri et al. (85) used advanced causal modelling (parametric g-formula) and found no strong association between exposure to non-persistent endocrine disruptors, including OP insecticides, and attention and inhibitory control.

## DISCUSSION

Our intention with this review was to complement previous reviews focused primarily on OPs (36, 86,87,88) or pyrethroids (7, 89, 90), with only a couple addressing multiple groups, including carbamates (13, 91). Even with our broadened scope, the majority of the studies encompassed by our review predominantly addressed exposure to OPs or, more broadly, to AChE inhibitors. This is not surprising considering that we did not limit our scope to the latest research, as OPs and carbamates have been in use the longest and are generally considered more toxic to humans than pyrethroids and neonicotinoids (16). Neonicotinoids were introduced in the 1990s, which may explain why their effects on children and adolescents have been examined in only two studies to date.

Biomonitoring was by far the most common method used to assess exposure. The same was concluded by Reed et al. (92) in a recent review of methodologies in studies assessing the association between pesticides exposure and neurodevelopment, behaviour, and/or cognition in children from birth to 18 years of age. Biomonitoring is commonly regarded as the “gold standard” in quantitative risk assessment, with its main advantage being that it reflects the degree of exposure regardless of the source and route (93, 94). In the analysed studies, exposure was most often assessed by measuring insecticide metabolite levels in urine. Although a discussion of the advantages and limitations of using specific versus nonspecific markers is beyond the scope of this review, it is important to note that one of the major criticisms is that knowing the levels of certain metabolites does not provide a complete picture of exposure (77). However, the main limitation of using urinary biomarkers to assess exposure to current non-persistent insecticides is that they metabolise rapidly and that their measurements generally reflect only acute, approximately 24-hour exposure (95). But this limitation does not necessarily apply to studies employing other biomonitoring methods such as measuring pesticide residues in hair or AChE inhibition in red blood cells or to studies incorporating additional indicators such as questionnaires or temporal or spatial proximity to pesticide application, which were the second most common method for establishing insecticide exposure.

Several researchers assessed cholinesterase (ChE) activity in blood samples as an indicator of exposure to insecticides which inhibit it irreversibly like OPs or reversibly, like carbamates (96). Most measured plasma or serum ChE, i.e., butyrylcholinesterase (BChE), and erythrocyte AChE, while a few utilised whole blood samples. Plasma/serum ChE (pseudocholinesterase, BChE) is typically regarded as an indicator of recent, acute exposure, whereas erythrocyte AChE reflects prolonged or chronic exposure and is a better indicator of effects on the nervous system (97).

Rodríguez-Carrillo et al. (74) used BDNF as a biomarker of neurodevelopmental impacts of exposure to several types of pesticides, measuring protein levels in serum and DNA methylation of the BDNF gene in whole blood. This provided a new insight supporting the mechanistic model that explains how OPs, pyrethroids, and carbamates act through different molecular and cellular pathways to ultimately inhibit release of BDNF and/or induce inflammatory processes in the brain. BDNF has many functions, including neuroprotection, cell development, synaptic plasticity, and modulation of synaptic interactions, which are all crucial for normal neurobehavioural functioning and development (98). Inhibited synthesis of BDNF at the system level is associated with impaired structural and functional connectivity, which could diminish neuropsychological functioning and development.

Thirty six of the 48 reviewed studies evidence some association between adverse neurobehavioural effects and childhood/adolescence insecticide exposure. Most have evaluated cognitive functioning, working memory in particular. At least half of them evidence adverse effects on executive functions, general intellectual functioning, processing speed/reaction time, sensory and motor function, attention and inhibitory control, verbal comprehension/crystallised intelligence, and fluid reasoning.

Our review reveals differences between studies investigating occupational and non-occupational insecticide exposure in the focus on outcome, study design, population, and geographic distribution. Occupational exposure studies mostly cover Africa and South America, reflecting the widespread use of child and adolescent labour in agriculture. These studies are mostly limited to smaller samples and male participants and mostly compare groups with higher- and lower-exposure risk. Their main interest is the cognitive function. The most consistently observed are the adverse effects on executive functions, working memory/immediate (short-term) memory, long-term memory, verbal comprehension, and processing speed, primarily linked to exposure to OPs, OP-containing mixtures, or non-specified insecticides. The effects on working/immediate/short-term memory and long-term memory/learning are more often reported for occupational than non-occupational exposure. Sensory and motor function follows a similar pattern.

In contrast, non-occupational (residential) exposure studies mostly cover the North and South America, Asia, and Europe and involve larger samples of early school to early adolescent-age participants. Unlike occupational studies, they most often include roughly matching numbers of boys and girls and explore the effects on emotional and behavioural functioning, mainly associated with exposure to OPs and pyrethroids. Adverse outcomes have primarily been reported for executive functions, processing speed, general/fluid intelligence, emotional and behavioural functioning, attention and inhibitory control, verbal comprehension, and fine motor skills.

Regardless of the differences, findings from both occupational and non-occupational studies underscore consistent associations between insecticide exposure and neurobehavioural impairments in children and adolescents. Even children not directly involved in agricultural work but exposed through environmental contamination (e.g., residential/temporal proximity to treated fields, home pesticide use, or dietary intake) still face significant risks of pesticide exposure-related changes in neurobehavioural functioning. The consistent effect on executive functions and frontal-lobe-dependent processes, which are still maturing throughout adolescence, suggests particular vulnerability of higher-order cognitive processes to insecticide-related neurotoxicity across both occupational and environmental exposure contexts.

It is quite difficult to quantify the severity or magnitude of the reported neurobehavioural effects, because the reviewed studies are rather heterogeneous in terms of exposure assessments, outcome measures, analytical approaches, and even age groups. However, we tried to get a general idea of the severity of reported insecticide exposure effects by limiting our analysis to four studies reporting the results for the same biomarker of exposure (AChE activity) and the same indicator [Digit Span Test (DST)] of working memory as the most evaluated neurobehavioural outcome. DST measures short-term and working memory on sequences of digits of increasing length that participants must repeat in the same (forward, DS-F) or reverse (backward, DS-B) order. Scores are usually reported as the longest correctly recalled sequence for each trial (DS-F, DS-B) or as the combined total score, although study (63) presented standardised, z-scores.

One Egyptian cross-sectional study (40) compared 100 younger (9–15 years) and older (16–18 years) male applicators with age-matched controls recruited from the same communities and schools. Applicators showed lower AChE activity (M=239.8 IU/L) than controls (M=283.1 IU/L; p<0.05), which significantly correlated with both DS-F and DS-B scores (r=0.40, p<0.05) after adjusting for age and work history. Younger applicators scored significantly lower than age-matched controls (DS-F=5.4 and DS-B=4.7 vs 6.1 and 5.5, respectively; p<0.05), with the effect sizes of around 0.3. Older applicators performed even more poorly (DS-F=4.0 and DS-B=3.5 vs 5.9 and 5.5, respectively; p<0.05), with large effect sizes of about 1.0.

A longitudinal study (60) of 84 Egyptian adolescents aged 12–21 years (sex not reported) showed a significant AChE inhibition between baseline and spraying season measurements, which correlated significantly with DS-F (r=0.20, p<0.05) after controlling for urinary TCPy, years of education, time of testing, job status, and testing station.

The Argentinian study (48) comparing 87 adolescents (sex not reported) aged 11–16 from three locations with different levels of pesticide exposure found no associations between AChE activity and DST performance but noted that adolescents in high- and moderate-exposure areas had significantly lower AChE activity (p<0.01), which was below the normal range (7120–11760 U/L). DST performance was also markedly worse in the high-exposure (M=11.15) than the moderate (M=12.60) and low-exposure groups (M=15.07), which is consistent with a dose-response pattern.

Finally, the third Egyptian study (63) of 41 adolescent males aged 12–18 years did not look into the relationship between AChE and DST; however, it reported no significant differences between applicators and controls in either the total DST z-score or AChE activity (25.4 U/g haemoglobin in applicators vs. 26.7 U/g haemoglobin in controls).

In summary, the evidence suggests that adolescents exposed to OPs tend to perform worse on DST, often in a dose-response fashion, and in some cases, deficits directly correlate with AChE inhibition. However, the heterogeneity of methods and different statistical power between designs still hinders precise estimation of the magnitude of effects.

Sleep as an outcome was investigated in only one, very recent study (75), which found that pyrethroid exposure was associated with increased sleep problems in male adolescents only. This lack of studies represents a major gap in the literature and underscores the need for future studies to include sleep variables. Sleep is a fundamental physiological need and plays a critical role in the development and maintenance of various vital physiological processes. During adolescence, some major changes in sleep patterns and architecture coincide with changes in secretory profiles of reproductive hormones and brain reorganisation patterns (99). Sleep health is a complex, multidimensional concept encompassing timing, duration, regularity, and other aspects of sleep quality, all of which can be influenced by various biological, psychosocial, contextual, and environmental factors (33, 34, 100, 101). Sleep problems in adolescents are recognised as public health concern, as inadequate sleep during this developmental period has been linked to negative outcomes in academic performance, neurobehavioural functioning, and physical health (33, 34). Among contextual factors, early school start times are consistently identified as one of the most pervasive contributors to inadequate sleep in adolescents (33, 34, 101). Poor sleep health is also being recognised as a vulnerability factor that can increase susceptibility to adverse effects from environmental and social contexts (102). Therefore, we find that incorporating assessments of sleep health into future study designs is important for a more comprehensive understanding of the potential risks associated with insecticide exposure.

In their systematic review of neurodevelopmental effect of prenatal and postnatal exposure to OPs, Sapbamrer and Hongsibsong (36) found that multiple studies consistently linked prenatal OP exposure to poorer neurobehavioural functioning and neurodevelopmental outcomes in school-age children (6–15 years of age), while evidence of the impact of postnatal exposure was limited, leading the authors to suggest that critical periods of vulnerability occur primarily during prenatal development. However, judging from the data gathered for this review, we believe that further large-scale longitudinal cohort studies are needed, particularly to elucidate the potential neurodevelopmental risks associated with exposure during peripubertal and pubertal periods. Given that these developmental stages involve significant hormonal and neural reorganisation, they may represent unique windows of heightened vulnerability. Moreover, as neurodevelopmental effects of pesticide exposure may only become apparent later in adolescence or even adulthood, extended follow-up periods are essential to accurately capture delayed consequences, should there be any.

With the exception of one study (76), pubertal status was neither analysed nor controlled for in studies, despite evidence suggesting that insecticide exposure around the time of puberty may accelerate or delay pubertal onset, potentially leading to long-term consequences for reproductive health and neuropsychological functioning (24, 103, 104). Chronological age is sometimes used – mainly in animal models – as an approximate indicator of pubertal development (29) and is one of the most common covariates in the studies included in this review. However, substantial variability in pubertal timing and progression in humans necessitates the use of more precise measures. Pubertal status can best be assessed through phenotypic or hormonal measures. Phenotypic assessments – objective (clinician-rated) or subjective (self- or parent-reported) – reflect the timing and effects of hormonal changes and include determining the stage of secondary sexual characteristics according to Marshall and Tanner (105, 106), which remains the clinical standard (29, 107). Measuring hormone concentrations in biological samples for markers of adrenarche (e.g., DHEA) and gonadarche (e.g., gonadotropins, testosterone) is particularly valuable for identifying earlier pubertal processes (29, 108).

In epidemiological studies specifically investigating the effects of exposure to non-persistent pesticides on pubertal timing, pubertal development was assessed using both phenotypic indicators and hormonal measures. In their systematic review, Castiello and Freire (24) found that exposure to different types of pesticides was associated with altered puberty timing in girls and/or boys in eight of thirteen studies. Exposure to multiple pesticides was generally associated with earlier puberty onset in girls and delayed puberty in boys. In girls, exposure to both OPs and pyrethroids was linked to delayed pubertal maturation (e.g., later menarche and breast development). In boys, OP exposure was linked to delayed pubertal development (e.g., smaller testicular volume, shorter penile length, lower serum testosterone levels), whereas exposure to pyrethroids was linked to early pubertal development (e.g., larger testicular size, and elevated LH and FSH levels).

Furthermore, instead of treating sex solely as a covariate, future studies should also examine sex differences in the neurobehavioural effects of exposure to insecticides, as only a few studies have directly examined sex differences, and our knowledge about this topic is limited. Some research has indicated sex-specific vulnerabilities to insecticide exposure. Some even suggested that sex difference could at least partly explain differences in the risk of developing various neurodevelopmental disorders (109). For a review of the effects of insecticide exposure on major neurodevelopmental disorders, readers are referred to other sources (6, 9, 110,111,112,113,114).

### Limitations of this review and of the reviewed studies

This review has several limitations that should be acknowledged. Although some of the reviewed studies evaluate symptoms related to ADHD (51, 52, 54, 73, 74, 82, 83, 115, 116), here we do not discuss them in detail. We did not include studies specifically examining neurodevelopmental disorders, since these conditions typically manifest themselves earlier in life and are influenced by multiple genetic and environmental factors. Also, current evidence indicates that prenatal exposure plays a more critical role in the aetiology of these disorders than postnatal/childhood exposure. We also do not discuss clinical neurological findings.

Another limitation is that we report exclusively on the effects of the insecticides of interest and overlook the potential contribution of other pesticide classes, such as herbicides and fungicides, even though they have been part of some studies included in this review.

Next, we did not calculate inter-rater agreement during title and abstract screening as a measure of the robustness of the screening process. However, we believe this is a minor limitation, considering that disagreements between screeners (authors) were rare, minimal, and readily resolved through discussion and consensus.

Then, there is the potential bias arising from the fact that studies reporting significant effects are more likely to be published (117), which may lead to an overestimation of the identified associations.

As we used narrative synthesis, this approach may have introduced additional (subjective) bias in interpretation. However, our conclusions are robust, because when synthesising evidence, we did not stratify results based on the specific exposure assessment methods or any other criteria such as age or sex, but we do report if the effect was observed only in boys of girls.

Furthermore, this review did not include a formal quality assessment of the studies, and all included studies were given equal weight in the conclusions.

Limitations to conclusions also arise from the limitations of the included studies. A great majority (n=31) rely on cross-sectional design, and exposure and outcomes are assessed as a single time point, which limits causal inference. A number of studies, especially those investigating occupational exposure, have small sample sizes, which begs the question of their statistical power.

Furthermore, many of the reviewed studies are limited to specific geographic regions and specific cohorts, which limits their generalisability to broader or more diverse populations.

The groups are quite heterogeneous across studies to enable consistent comparison, as they are more centred around location or occupation than on direct exposure measurement. What is more, not all control groups are truly unexposed. For example, Fiedler et al. (59) report unexpected findings of significantly higher urinary OP metabolite levels during low than high pesticide-use season in both children living on rice farms (higher risk group) and children living on a shrimp farm (control group).

Another methodological limitation is the heterogeneity in exposure assessment, ranging from biomarkers to proximity-based estimates, which also complicates comparison and synthesis of the findings. The timing of exposure assessment also varies greatly across studies. Most assess either current or cumulative exposure up to the time of neurobehavioural testing, spanning from several days to several years. In the CHAMACOS cohort studies, however, there was a time gap between the exposure assessment period and neurobehavioural assessment, where researchers report exposure accumulated between six months and five years of age (54, 70, 115, 116).

Similarly, assessments of neurobehavioural functioning vary considerably across studies, particularly in the evaluation of cognitive functions. To address this variability, we attempted to identify the primary cognitive function or process targeted by each assessment instrument and then categorised them into ten overarching domains (Table 1), so that we could draw robust conclusions from findings obtained using diverse instruments.

As mentioned earlier, many studies use broad or even poorly defined age groups, resulting in substantial variability in age ranges. We find this also impeding our effort to draw conclusions about insecticide effects during puberty and adolescence.

In general, the heterogeneity of the reviewed studies makes it difficult to quantify the severity or magnitude of the reported insecticide effects on neurobehavioural functioning in puberty and adolescence. It is important to note, however, that all four European studies demonstrated high methodological quality. They were conducted on large, well-characterised birth cohorts and used sophisticated exposure assessment methods, including biomarkers, spatial and temporal proximity indicators, and, in the case of the HELIX project (55, 67), comprehensive exposome-wide analyses adjusted for multiple co-exposures.

## CONCLUSION

In conclusion, although some findings are mixed or inconclusive, the prevailing evidence suggests an association between childhood/adolescence insecticide exposure and adverse neurobehavioural effects in 8–20-year-olds, most of them on cognitive functions. Studies of occupational exposure report the effects on working memory/immediate (short-term) memory, long-term memory/learning, and sensory and motor function more frequently and associate them with OPs, OP-containing mixtures, or unspecified insecticides. Non-occupational studies, in turn, most often report adverse effects on executive functions, attention and inhibitory control, processing speed, general and fluid intelligence, and emotional and behavioural functioning, predominantly associated with exposure to OPs and pyrethroids.

However, the reviewed studies vary substantially in design, sample size, comparison groups, exposure proxies, neurobehavioural assessment tools, and covariate adjustment, which limits the strength of conclusions that can be drawn. Future studies should combine longitudinal and multidisciplinary approach to elucidate the effects of insecticide exposure on sleep and neurobehavioural functions during the developmentally dynamic periods of puberty and adolescence. This field of research would greatly benefit from cohort studies which follow children from the peripubertal age through adolescence with repeated exposure assessments and which explicitly account for pubertal development and sex differences. Furthermore, the use of standardised test batteries for neurobehavioural assessment that include sleep measures is essential to enable comparability across studies and improve measurement precision and characterisation of potential risks associated with insecticide exposure.

Overall, findings from the reviewed studies suggest that both occupational and non-occupational pesticide exposure warrant concern for adolescent neurodevelopment and sleep, but more rigorous and methodologically consistent investigations are needed.
